# Robust Multi-Frame Adaptive Optics Image Restoration Algorithm Using Maximum Likelihood Estimation with Poisson Statistics

**DOI:** 10.3390/s17040785

**Published:** 2017-04-06

**Authors:** Dongming Li, Changming Sun, Jinhua Yang, Huan Liu, Jiaqi Peng, Lijuan Zhang

**Affiliations:** 1 School of Information Technology, Jilin Agricultural University, Changchun 130118, China; ldm0214@163.com (D.L.); huan_liu_2017@sohu.com (H.L.); 18404317389m@sina.cn (J.P.); 2 School of Opto-Electronic Engineering, Changchun University of Science and Technology, Changchun 130022, China; yangjh@cust.edu.cn; 3 CSIRO Data61, PO Box 76, Epping, NSW 1710, Australia; changming.sun@csiro.au; 4 College of Computer Science and Engineering, Changchun University of Technology, Changchun 130012, China

**Keywords:** atmospheric turbulence, image restoration, adaptive optics, blind deconvolution, maximum likelihood, frame selection

## Abstract

An adaptive optics (AO) system provides real-time compensation for atmospheric turbulence. However, an AO image is usually of poor contrast because of the nature of the imaging process, meaning that the image contains information coming from both out-of-focus and in-focus planes of the object, which also brings about a loss in quality. In this paper, we present a robust multi-frame adaptive optics image restoration algorithm via maximum likelihood estimation. Our proposed algorithm uses a maximum likelihood method with image regularization as the basic principle, and constructs the joint log likelihood function for multi-frame AO images based on a Poisson distribution model. To begin with, a frame selection method based on image variance is applied to the observed multi-frame AO images to select images with better quality to improve the convergence of a blind deconvolution algorithm. Then, by combining the imaging conditions and the AO system properties, a point spread function estimation model is built. Finally, we develop our iterative solutions for AO image restoration addressing the joint deconvolution issue. We conduct a number of experiments to evaluate the performances of our proposed algorithm. Experimental results show that our algorithm produces accurate AO image restoration results and outperforms the current state-of-the-art blind deconvolution methods.

## 1. Introduction

Because of the turbulent flow field, an imaging system with adaptive optics (AO) is interfered by heat, thermal radiation, and image transmission. The observed image contains pointing jitters and is fuzzy, and this effect is known as the aero optical effect [1,2]. However, the AO correction within the system is often only partial, and the long-exposure images must be restored by postprocessing, where a deconvolution is required for reaching the diffraction limit. The AO images in a real system are usually represented by the convolution of an ideal image with a point spread function (PSF) [3]. The AO image is also contaminated by other noises, such as read-out, photon counting, multiplicative, and compression noise [4,5]. In most practical applications, however, finding the real PSF is impossible and an estimation must be carried out. Because the observed AO image is corrupted by various sources of noise, the estimation of both the object image and the PSF should be carried out at the same time, and this process is called blind deconvolution [6].

Blind deconvolution is also very sensitive to the noise present in the observed images [7,8]. To handle this problem, researchers have been making efforts in developing AO image restoration algorithms under various conditions. Using a total variation (TV) prior on the variational Bayesian blind deconvolution algorithm (VBBD-TV algorithm), Babacan et al. [9] developed an algorithm which shows good restoration performance for total variation based blind deconvolution and parameter estimation utilizing a variational framework. Katsaggelos and Lay proposed a maximum likelihood (ML) blur identification and image restoration method using the Expectation-Maximization algorithm (ML-EM algorithm) [10], which uses no knowledge about the type of distortion or its support region during iteration.

Zhu et al. presented an adaptive algorithm for image restoration using combined penalty functions (CPF-adaptive algorithm) [11]. This algorithm is adaptive and is used to estimate the smoothing and roughness parameters. The advantage of this method is that it is able to remove noise at the same time when restoring the image information around edges.

Zhang et al. proposed a multi-frame iterative blind deconvolution algorithm based on an improved expectation maximization algorithm for AO image restoration (RT-IEM algorithm) [12], which represents a more complex function to minimize. This algorithm contains a cost function for the joint-deconvolution of multi-frame AO images, and it estimates the regularization terms. The major limitation for this algorithm is its computational speed. Robust blind deconvolution methods optimize a data fidelity term, which can be stabilized by some additional regularization terms. Yap and Guan [13] proposed an approach for adaptive image regularization based on a neural network and a hierarchical cluster model. The main advantage of the method is that the empirical relationship between the optimized regularization vector and the local perception measure can be reused in the restoration of other degraded images. The major limitation for the algorithm is its convergence speed.

Although a number of blind deconvolution methods have been proposed, a good knowledge of the PSF is always desirable in order to achieve an accurate restoration. For example, Deforest et al. [14] modeled the scattering portion of the transition region and coronal explorer (TRACE) PSF as the sum of a measured diffraction pattern and a circularly symmetric scattering profile. In addition to constructing a parametric model of the PSF using the specification instrument, Poduval et al. modeled the PSF with a diffraction kernel and an isotropic scattering term representing stray light [15]. In these works, the parametric methods have the limitation that they depend on a particular instrument and the PSF models need to be available [16].

Our goal in this paper is to propose a new deconvolution scheme based on our frame selection method and the initial PSF estimation approach. The frame selection method is used to choose the better quality images from the multi-frame short exposure image sequence, which is initially corrected by the AO system. We take the variance of the AO image as the evaluation method for frame selection. The turbulence effect of an AO imaging system refers to the fact that when a reference star is regarded as the center for wavefront detection, the fluctuation of wavefront is no longer consistent with the detected wavefront for the regions that are beyond an isoplanatic angle. If this effect exists for an imaging process, it affects the restoration of AO images. Therefore, we develop the method for initial PSF estimation, which is a parametric method based on the physics of the instrument, and we provide a more precise PSF model containing some instrument’s properties. The initial estimation of the PSF model takes into account the a priori information on the object and the AO system parameters. A theoretical expression of the PSF is derived. The proposed PSF initial estimation is also evaluated by sensitivity analysis techniques and an error analysis is carried out based on simulation results (with and without noise).

The outline of our paper is as follows. Section 2 describes the frame selection method and the PSF model. Section 3 presents our new algorithm for joint blind deconvolution based on Poisson distribution and gives several methods for improved ML estimation and regularization. The restoration algorithm is applied to real AO images and the results are presented in Section 4. Finally in Section 5 we conclude.

## 2. Frame Selection Method and PSF Model

### 2.1. AO Image Degradation Model

Affected by atmospheric turbulence, observed images from astronomical imaging and remote sensing often suffer from image quality degradation. The readout noise satisfies an additive Gaussian model [17], and the degradation process can be represented as

(1) i(x,y)=o(x,y)⊗h(x,y)+c(x,y),(x,y)∈Ω,

where (x,y) is the spatial coordinate in the image, o(x,y) is the original image, h(x,y) refers to the PSF, i(x,y) represents the degraded image acquired by the image sensor, c(x,y) is the noise, Ω is the region of the object image, and ⊗ stands for the convolution operation.

A multi-frame AO image degradation model can be represented as

(2) $$i_{k}\left(x,y\right)=o\left(x,y\right)⊗h_{k}\left(x,y\right)+c\left(x,y\right),\;1<k\leq M,$$

where $i_{k}\left(x,y\right)$ denotes the *k*th frame of the observed AO images, $h_{k}\left(x,y\right)$ represents its corresponding PSF, and *M* is the total number of frames. In this paper, we use the sequence of the degraded multi-frame images $i_{k}$ (k=1,2,…,M) of the same object to restore the original image *o*.

### 2.2. Frame Selection Technique Based on Variance

In an experiment, the system can collect thousands of images for the same object. Interfered by atmospheric turbulence with various strengths, the AO system compensates the turbulence effects, and the image restoration results are directly affected by each frame. We select better quality frames in a sequence of images for image restoration [18].

A frame selection technique is used to select the high quality images from the multi-frame images that are initially corrected by an AO system. There are a number of evaluation criteria that can be used to evaluate the quality of degraded images. In this paper, we use the variance of an image as the frame evaluation method. The variance of the *k*th frame of an observed degraded image $i_{k}\left(x,y\right)$ can be calculated by

(3) $$S_{k}^{2}=\frac{1}{N_{1}N_{2}}\sum_{x=1}^{N_{1}}\sum_{y=1}^{N_{2}}{\left(i_{k}\left(x,y\right)-\mu_{k}\right)}^{2},$$

where $N_{1}$ and $N_{2}$ represent the width and height of the observed image, respectively, and $\mu_{k}$ refers to the mean of an observed image $i_{k}\left(x,y\right)$

(4) $$\mu_{k}=\frac{1}{N_{1}N_{2}}\sum_{x=1}^{N_{1}}\sum_{y=1}^{N_{2}}i_{k}\left(x,y\right).$$

The mean of variance $S_{\mathrm{mean}}^{2}$ of the multi-frame observed images is defined as

(5) $$S_{\mathrm{mean}}^{2}=\frac{1}{M}\sum_{k=1}^{M}S_{k}^{2}.$$

The constraint for frame selection is defined as

(6) $$|S_{k}^{2}-S_{\mathrm{mean}}^{2}|<T,$$

where T is the threshold for frame selection. In this method, the variance value $S_{k}^{2}$ of the degraded image should be close to the mean of variance $S_{\mathrm{mean}}^{2}$. Note that $S_{k}^{2}$ can be higher than $S_{\mathrm{mean}}^{2}$ or lower than it. We only keep those images with variance values in the middle. Images with too low variance will be removed, and images with too high ones will also be removed.

The variance for the observed multi-frame images is defined as a sequence of data samples $\left\{S_{1}^{2},S_{2}^{2},\ldots ,S_{M}^{2}\right\}$, and the variance $\sigma_{s}^{2}$ of the data samples is

(7) $$\sigma_{s}^{2}=\frac{1}{M}\sum_{k=1}^{M}{\left(S_{k}^{2}-S_{\mathrm{mean}}^{2}\right)}^{2}.$$

In the frame selection method, we choose the threshold *T* according to the variance $\sigma_{s}^{2}$. In the frame selection process, we have carried out a lot of experiments and chosen different *T* values for obtaining high quality images from the collected thousands of degraded AO images for the same object. Finally, we found that the threshold T is approximately 2–3 times of $\sigma_{s}^{2}$, which can obtain better frames in a sequence of degraded AO images.

The frame selection process is based on an iterative algorithm. That is, when a better frame has been selected, the threshold *T* is recalculated. Then, the remaining frames will be checked by this method until the constraint is satisfied. The frame selection algorithm is shown in Algorithm 1.

**Algorithm 1** Frame selection**Step 1**: Initialize and calculate the variance set $\left\{S_{1}^{2},S_{2}^{2},\ldots ,S_{M}^{2}\right\}$ for $\{i_{k}\left(x,y\right)\},k=1,2,\ldots ,M$;**Step 2**: Calculate the following with iteration number j=1,2,…,MaxIterations
According to Equation (5), calculate the mean value $S_{\mathrm{mean}}^{2}$ of sequence $\left\{S_{1}^{2},S_{2}^{2},\ldots ,S_{M}^{2}\right\}$;According to Equation (7), calculate the variance $\sigma_{s}^{2}$ of sequence $\left\{S_{1}^{2},S_{2}^{2},\ldots ,S_{M}^{2}\right\}$;Set the threshold $T=2.6\sigma_{s}^{2}$;Check the constraint as in Equation (6);Update the sequence $\left\{i_{k}\left(x,y\right)\right\}$ after frame selection. If $\left\{i_{k}\left(x,y\right)\right\}$ no longer changes, then go to **Step 3**;Increase *j*;**Step 3**: If the number of iterations is MaxIterations or if $i_{k}\left(x,y\right)$ does not change, output the selected images $\left\{i_{k}\left(x,y\right)\right\}$ and finish; otherwise, go to **Step 2**.


In Algorithm 1, the threshold $T=2.6\sigma_{s}^{2}$ was estimated experimentally by running the frame selection method with different *T*s and selecting the one with the most visually acceptable results.

### 2.3. PSF Model

According to Veran et al. [19], the wavefront phase that is corrected by the AO system is still a quasi-stationary random process. The optical transfer function (OTF) of an AO system is defined as

(8) $$H_{a}\left(u\right)=\exp(-\frac{C_{a}\left(\lambda fu\right)}{2}),$$

where *f* is the focal length of the imaging system, $C_{a}\left(\lambda fu\right)$ is the phase structure function of the wavefront, and its calculation formula can be expressed as

(9) $$C_{a}\left(\lambda fu\right)=C_{a}\left(\Delta z\right)=\langle {\left(\phi_{\mathrm{res}}\left(z\right)-\phi_{\mathrm{res}}\left(z+\Delta z\right)\right)}^{2}\rangle ,$$

where 〈·〉 denotes the expectation over turbulence realizations, *z* is a space vector within the telescope pupil, Δz is a space interval vector within the telescope pupil, and $\phi_{\mathrm{res}}\left(z\right)$ denotes the residual wavefront phase after an AO system correction.

According to the analysis in [20,21], the residual wavefront phase structure function $C_{a}\left(\Delta z\right)$ is the sum of an isoplanatic and an anisoplanatic structure functions, which is defined as

(10) $$C_{a}\left(\Delta z\right)=C_{0}\left(\Delta z\right)+\tilde{C}\left(\Delta z,\theta \right),$$

where θ is the off-axis angle interval, $C_{0}\left(\Delta z\right)$ represents the on-axis isoplanatic structure function, and $\tilde{C}\left(\Delta z,\theta \right)$ represents the anisoplanatic structure function, which is defined as

(11) $$\begin{array}{cc} \tilde{C}\left(\Delta z,\theta \right)= & \langle {\left(\phi_{\mathrm{res},\theta }\left(z\right)-\phi_{\mathrm{res},\theta }\left(z+\Delta z\right)\right)}^{2}\\ & -{\left(\phi_{\mathrm{res},0}\left(z\right)-\phi_{\mathrm{res},0}\left(z+\Delta z\right)\right)}^{2}\rangle . \end{array}$$

According to Zernike polynomial theory and assuming that an AO system can fully compensate for the first *n* order Zernike polynomials, the residual wavefront phase of isoplanatic $\phi_{\mathrm{res},0}\left(z\right)$ and residual wavefront phase of anisoplanatic $\phi_{\mathrm{res},\theta }\left(z\right)$ are expressed as [22]

(12) $$\begin{array}{c} \phi_{\mathrm{res},\theta }\left(z\right)=2\pi \left(\sum_{j=1}^{n}\left(a_{j}\left(\theta \right)-a_{j}\left(0\right)\right)Z_{j}\left(z\right)+\sum_{j=n+1}^{\infty }a_{j}\left(\theta \right)Z_{j}\left(z\right)\right),\\ \phi_{\mathrm{res},0}\left(z\right)=2\pi \sum_{j=n+1}^{\infty }a_{j}\left(0\right)Z_{j}\left(z\right), \end{array}$$

where $a_{j}\left(0\right)$ and $a_{j}\left(\theta \right)$ represent the coefficients of the on-axis and off-axis Zernike polynomial, respectively, and $Z_{j}\left(z\right)$ is the *j*th order Zernike polynomial.

Under the anisoplanatic situation, the OTF of the imaging system is defined as

(13) $$\tilde{H}\left(u,\theta \right)=\exp(-\frac{\tilde{C}\left(\lambda fu,\theta \right)}{2}).$$

Similarly, modifying Equation (8) and the atmospheric environment, an OTF of an AO system is defined as

(14) $$H_{a}\left(u\right)=H_{0}\left(u\right)\tilde{H}\left(u,\theta \right),$$

where $H_{0}\left(u\right)$ is an OTF for the anisoplanatic effect.

Therefore, an OTF of the whole AO system is

(15) $$H(u,\theta )=H_{a}\left(u\right)H_{\mathrm{tel}}\left(u\right)=H_{0}\left(u\right)\tilde{H}\left(u,\theta \right)H_{\mathrm{tel}}\left(u\right),$$

where $H_{\mathrm{tel}}\left(u\right)$ is the telescope diffraction-limited OTF, which is [20]

(16) $$H_{\mathrm{tel}}\left(u\right)=\frac{2}{\pi }\mathrm{circ}\left(\frac{u}{D}\right)\left(\arccos\left(\frac{u}{D}\right)-\frac{u}{D}\sqrt{1-\frac{u^{2}}{D^{2}}}\right),$$

where *D* is the telescope diameter, and $\mathrm{circ}\left(\frac{u}{D}\right)$ will return a circle mask array with a circle of radius $\frac{u}{D}$ and the default array size is 128×128 with a central location at the middle of the array.

Modifying Equation (11) according to [11], we obtain the expression for the anisoplanatic structure function, which is

(17) $$\tilde{C}\left(\Delta z,\theta \right)=\sum_{k=1}^{n}\sum_{j=1}^{\infty }2\left(-\Gamma_{a}\left(\theta \right)+\Gamma_{a}\left(0\right)\right)Z_{k,j}\left(\Delta z\right),$$

where $\Gamma_{a}\left(\theta_{1}-\theta_{2}\right)=\langle a_{k}\left(\theta_{1}\right)a_{j}\left(\theta_{2}\right)\rangle$, $\Gamma_{a}\left(\theta \right)$ is the angular correlation function between coefficients $a_{k}$ and $a_{j}$ of the Zernike polynomial, and $Z_{k,j}\left(\Delta z\right)$ are functions defined as [21]

(18) $$Z_{k,j}\left(\Delta z\right)=\frac{\int \left(Z_{k}\left(z\right)-Z_{k}\left(z+\Delta z\right)\right)\left(Z_{j}\left(z\right)-Z_{j}\left(z+\Delta z\right)\right)dz}{\int P(z)P(z+\Delta z)dz},$$

where P(z) is the telescope pupil function.

Based on Equations (13), (15) and (17), the OTF of an AO imaging system can be calculated. Therefore, the OTF can be transformed to PSF by the inverse Fourier transform, which is

(19) $$PSF=F^{-1}\left\{H\left(u,\theta \right)\right\}.$$

In this paper, we adopt the above method to rebuild the AO-PSF estimated model as an initial estimation for the AO image restoration. Considering that the isoplanatic angle is $2^{\prime \prime }$, it is equivalent to a square isoplanatic area with $2^{\prime \prime }\times 2^{\prime \prime }$. Assuming that the PSF is with circular symmetry, we build the PSF model when the field-of-view is $10^{\prime \prime }$, and the grid is 5×5. Based on Equation (1) for the image deconvolution reconstruction, we will describe our AO image restoration method in Section 3.

## 3. Joint Blind Deconvolution Algorithm Based on Poisson Distribution

### 3.1. Estimators with Poisson Statistics

A blind deconvolution algorithm is to estimate both the object and the PSF at the same time. The noise in an AO image is mainly photon noise that has a Poisson distribution. However, an AO astronomy image is dominated by a quite homogeneous background [23,24]. The intensity convolution based on the Shell theorem describes the non-coherent imaging system, the object image {o(a,b),(a,b)∈Ω} which is a nonnegative function, and the PSF $\left\{h_{k}\left(\left(x,y\right)|\left(a,b\right)\right),\left(x,y\right)\in D\right\}$ (*D* is the region of the observed image) which is affected by atmospheric turbulence. Then, we define the object intensity $e_{k}\left(x,y\right)$ at point (x,y) in the *k*th frame as [25]

(20) $$e_{k}\left(x,y\right)=\sum_{(a,b)\in \Omega }h_{k}\left(\left(x,y\right)|\left(a,b\right)\right)o\left(x,y\right)=\sum_{(a,b)\in \Omega }h_{k}\left(x-a,y-b\right)o\left(a,b\right),\;1<k\leq M,$$

where (x,y)∈D, and Ω is the region of the object image. In fact, the value of $e_{k}$ cannot be detected perfectly because it is always contaminated by some noise.

Considering the cases where the degraded image with atmosphere turbulence has noise, the intensity $e_{k}\left(x,y\right)$ at each point (x,y) in the observed image is a random variable that follows an independent Poisson distribution. The ML estimator based on Poisson distribution can be expressed as [23,25]

(21) $$p\left(i_{k}\left(x,y\right)|o,h_{k}\right)=\frac{\sum_{(x,y)\in \Omega }{\left(o\left(x,y\right)h_{k}\left(x,y\right)\right)}^{i_{k}\left(x,y\right)}}{\prod_{k=1}^{M}i_{k}\left(x,y\right)}\times \exp\left(-\sum_{(x,y)\in \Omega }o\left(x,y\right)h_{k}\left(x,y\right)\right).$$

The intensity $e_{k}\left(x,y\right)$ in Equation (20) is taken into account in the calculation of the ML estimator as in Equation (21), which is

(22) $$p(i_{k}\left(x,y\right)|o,h_{k})=\frac{{\left(e_{k}\left(x,y\right)\right)}^{i_{k}\left(x,y\right)}\exp\left(-e_{k}\left(x,y\right)\right)}{\prod_{k=1}^{M}i_{k}\left(x,y\right)}.$$

Assuming that the pixels of the observed image are independent from each other [15], the joint likelihood function can be defined as

(23) $$p(i_{k}\left(x_{1}x_{2}\cdots x_{N_{1}},y_{1}y_{2}\cdots y_{N_{2}}\right)|o,h_{k})=\prod_{(x,y)\in \Omega }\frac{{\left(e_{k}\left(x,y\right)\right)}^{i_{k}\left(x,y\right)}\exp\left(-e_{k}\left(x,y\right)\right)}{\prod_{k=1}^{M}i_{k}\left(x,y\right)}.$$

### 3.2. Algorithm Implementation for AO Image Restoration

To estimate $\hat{o}$ and $\hat{h}$, an iterative deconvolution algorithm can be used. The $\hat{o}$ and $\hat{h}$ are based on the maximum likelihood estimation criterion, and the cost function can be written as

(24) $$J_{1}\left(o,h_{k}\right)=\arg\min_{i_{k}}\ln\left(p\left(i_{k}\left(x_{1}x_{2}\cdots x_{N_{1}},y_{1}y_{2}\cdots y_{N_{2}}\right)|o,h_{k}\right)\right)$$

Taking the logarithm on both sides of Equation (23), the log-likelihood function is defined as

(25) $$\begin{array}{c} \ln(p\left(i_{k}\left(x_{1}x_{2}\cdots x_{N_{1}},y_{1}y_{2}\cdots y_{N_{2}}\right)|o,h_{k}\right))\\ =-\sum_{(x,y)\in \Omega }e_{k}+\sum_{(x,y)\in \Omega }i_{k}\log e_{k}-\sum_{(x,y)\in \Omega }\log\prod_{k=1}^{M}i_{k}\left(x,y\right). \end{array}$$

It is assumed that the *M* observed images $\{i_{1}\left(x_{1}x_{2}\ldots x_{N1},y_{1}y_{2}\ldots y_{N2}\right)$, $i_{2}\left(x_{1}x_{2}\ldots x_{N1},y_{1}y_{2}\ldots y_{N2}\right)$, ⋯, $i_{M}(x_{1}x_{2}\ldots x_{N1}$, $y_{1}y_{2}\ldots y_{N2})\}$ are statistically independent [26], and the log-likelihood function of the multi-frame joint estimator is expressed as

(26) $$\ln(p\left(\left\{i_{k}\right\}|o,\left\{h_{k}\right\}\right)=-\sum_{k=1}^{M}\sum_{(x,y)\in \Omega }e_{k}+\sum_{k=1}^{M}\sum_{(x,y)\in \Omega }i_{k}\log e_{k}+b.$$

The last term of Equation (25) is a constant, and we represent it as *b* in Equation (26).

Based on Equations (25) and (26), we modify the cost function $J_{1}\left(o,h_{k}\right)$ as

(27) $$J_{1}^{\prime }\left(o,\left\{h_{k}\right\}\right)=\arg\min_{i_{k}}\ln\left(p\left(\left\{i_{k}\right\}|o,\left\{h_{k}\right\}\right)\right).$$

However, it has been shown that the restoration algorithm does not converge to the solution because the noise is amplified during iterations [25]. To avoid excessive noise amplification, regularization on the cost function is required. We combine Equation (27) with regularization parameters to establish the cost function for joint deconvolution for multi-frame AO images, which is

(28) $$J_{\mathrm{multi}}\left(o,\left\{h_{k}\right\}\right)=J_{1}^{\prime }\left(o,\left\{h_{k}\right\}\right)+\frac{|\nabla \hat{o}\left(x,y\right)|}{\gamma (\nabla v)},$$

where the second term is a regularization term that can be defined as

(29) $$|\nabla \hat{o}\left(x,y\right)|=\sqrt{{\left(\nabla o_{x}\left(x,y\right)\right)}^{2}+{\left(\nabla o_{y}\left(x,y\right)\right)}^{2}}$$

and

(30) $$\gamma (\nabla v)=\exp(-\frac{{\left|\nabla v\right|}^{2}}{2\xi^{2}}),$$

where γ(∇v) has a variable regularization coefficient associated with the gradient of each point with 0<γ(∇v)≤1 [12,27]; parameter ξ is the smoothing coefficient; and ∇v is the gradient for point v(x,y), where *v* is a gray pixel value.

In order to minimize the cost function, Equation (28) can be differentiated with respect to o(x,y) and $h_{k}\left(x,y\right)$, and then make the differentiation equal to zero, which is

(31) $$\frac{\partial J_{\mathrm{multi}}\left(o,\left\{h_{k}\right\}\right)}{\partial o(x,y)}=0,$$

(32) $$\frac{\partial J_{\mathrm{multi}}\left(o,\left\{h_{k}\right\}\right)}{\partial h_{k}\left(x,y\right)}=0.$$

In order to take the o(x,y) derivative easily, $e_{k}\left(x,y\right)$ can be formulated by Equation (20), and then brought into Equation (26). According to Equations (26), (28), and (31), the derivation process is

(33) $$\begin{array}{c} \frac{\partial J_{\mathrm{multi}}\left(o,\left\{h_{k}\right\}\right)}{\partial o(x,y)}=\frac{\partial J_{1}^{\prime }\left(o,\left\{h_{k}\right\}\right)}{\partial o(x,y)}+\frac{\partial (\frac{|\nabla \hat{o}\left(x,y\right)|}{\gamma (\nabla v)})}{\partial o(x,y)}\\ =\frac{\partial (-\sum_{k=1}^{M}\sum_{(x,y)\in \Omega }e_{k}+\sum_{k=1}^{M}\sum_{(x,y)\in \Omega }i_{k}\log e_{k}+b)}{\partial o(x,y)}+\frac{1}{\beta \gamma (\nabla v)}\times \mathrm{div}\left(\frac{\nabla \hat{o}\left(x,y\right)}{|\nabla \hat{o}\left(x,y\right)|}\right)\\ =-\sum_{k=1}^{M}\sum_{(x,y)\in \Omega }h_{k}\left(x,y\right)+\sum_{k=1}^{M}\sum_{(x,y)\in \Omega }i_{k}\left(x,y\right)\frac{h_{k}\left(x,y\right)}{\sum_{(x,y)\in \Omega }h_{k}\left(x,y\right)o\left(x,y\right)}\\ +\frac{1}{\beta \gamma (\nabla v)}\times \mathrm{div}\left(\frac{\nabla \hat{o}\left(x,y\right)}{|\nabla \hat{o}\left(x,y\right)|}\right)\\ =0. \end{array}$$

With the condition of image energy conservation, the sum value of each frame PSF of the turbulence degraded images is 1, namely $\sum_{k=1}^{M}\sum_{(x,y)\in \Omega }h_{k}\left(x,y\right)=1$. According to conservation of energy, the image energy before and after degradation remains unchanged, that is

(34) $$\frac{1}{1-\frac{1}{\beta \gamma (\nabla v)}\mathrm{div}\left(\frac{\nabla \hat{o}\left(x,y\right)}{|\nabla \hat{o}\left(x,y\right)|}\right)}\sum_{k=1}^{M}\sum_{(x,y)\in \Omega }i_{k}\left(x,y\right)\frac{h_{k}\left(x,y\right)}{\sum_{(x,y)\in \Omega }h_{k}\left(x,y\right)o\left(x,y\right)}=1.$$

Therefore, an iterative relationship can be established as follows:

(35) $${\hat{o}}^{\left(n+1\right)}\left(x,y\right)={\hat{o}}^{n}\left(x,y\right)\sum_{k=1}^{M}\sum_{(x,y)\in \Omega }i_{k}\left(x,y\right)\frac{{\hat{h}}_{k}^{n}\left(x,y\right)}{\sum_{(x,y)\in \Omega }{\hat{h}}_{k}^{n}\left(x,y\right){\hat{o}}^{n}\left(x,y\right)}\;\times \frac{1}{1-\frac{1}{\beta \gamma (\nabla v)}\mathrm{div}\left(\frac{\nabla {\hat{o}}^{n}\left(x,y\right)}{|\nabla {\hat{o}}^{n}\left(x,y\right)|}\right)},$$

where ${\hat{o}}^{n}\left(x,y\right)$ and ${\hat{h}}_{k}^{n}\left(x,y\right)$ are the results of the *n*th iteration.

In order to take the $h_{k}\left(x,y\right)$ derivative easily, $e_{k}\left(x,y\right)$ can be formulated by Equation (20). According to Equations (28) and (32), the solution is

(36) $$\begin{array}{c} \frac{\partial J_{\mathrm{multi}}\left(o,\left\{h_{k}\right\}\right)}{\partial h_{k}\left(x,y\right)}=\frac{\partial J_{1}^{\prime }\left(o,\left\{h_{k}\right\}\right)}{\partial h_{k}\left(x,y\right)}+\frac{\partial (\frac{|\nabla \hat{o}\left(x,y\right)|}{\gamma (\nabla v)})}{\partial h_{k}\left(x,y\right)}\\ =\frac{\partial (-\sum_{k=1}^{M}\sum_{(x,y)\in \Omega }e_{k}+\sum_{k=1}^{M}\sum_{(x,y)\in \Omega }i_{k}\log e_{k}+b)}{\partial h_{k}\left(x,y\right)}+0\\ =-\sum_{(x,y)\in \Omega }o\left(x,y\right)+\sum_{(x,y)\in \Omega }i_{k}\left(x,y\right)\frac{o(x,y)}{\sum_{(x,y)\in \Omega }h_{k}\left(x,y\right)o\left(x,y\right)}\\ =0. \end{array}$$

Because the object image is within the support region of the observed image, the original object image o(x,y) is normalized in advance, namely $\sum_{(x,y)\in \Omega }o\left(x,y\right)=1$. Therefore, within the support region for the observed image, the energy sum value for the original object images is 1, which is

(37) $$\sum_{(x,y)\in \Omega }i_{k}\left(x,y\right)\frac{o(x,y)}{\sum_{(x,y)\in \Omega }h_{k}\left(x,y\right)o\left(x,y\right)}=1.$$

When the object ${\hat{o}}^{\left(n+1\right)}\left(x,y\right)$ is estimated by Equation (35), we can build the iterative relationship of the new PSF, ${\hat{h}}_{k}^{\left(n+1\right)}\left(x,y\right)$, which is

(38) $$\begin{array}{c} {\hat{h}}_{k}^{\left(n+1\right)}\left(x,y\right)={\hat{h}}^{n}\left(x,y\right)\sum_{(x,y)\in \Omega }\left\{{\hat{o}}^{n}\left(x,y\right)\frac{i(x,y)}{\sum_{(x,y)\in \Omega }{\hat{h}}^{n}\left(x,y\right){\hat{o}}^{n}\left(x,y\right)}\right\},\\ {\hat{h}}^{\left(n+1\right)}\left(x,y\right)=\frac{{\hat{h}}^{n}\left(x,y\right)}{\sum_{(x,y)\in \Omega }{\hat{h}}_{k}^{n}\left(x,y\right)}, \end{array}$$

where β is a constant.

In summary, the object image $\hat{o}$ and the estimation of the PSF model, $\hat{h}$, are obtained by multiple iterations of Equations (35) and (38). The specific implementation steps for our algorithm are summarized in Algorithm 2.

**Algorithm 2** Steps for our proposed restoration algorithm**Step 1**: Initialize operation. According to the method described in Section 2.2, the *M* frames of images $\left(i_{1},i_{2},\ldots ,i_{M}\right)$ are obtained with our frame selection technique. Then, the initial object image is ${\hat{o}}_{0}=\left(i_{1}+i_{2}+\cdots +i_{M}\right)/M$;**Step 2**: Obtain the initial estimation of the PSF model, ${\hat{h}}_{0}$, according to Equation (19), with the algorithm described in Section 2.3;**Step 3**: Calculate parameter values. β is a constant. According to the scheme and formula described in Section 3.2, estimate the regularization parameters γ(∇x);**Step 4**: Iterate through *j*=1, 2, *…*, MaxIteration (MaxIteration = 200 or 300):
The inner loop count variable of PSF h_count=0;The iteration process of PSF estimation, *p*=0, 1, *…*, Max_count(a)Complete the PSF estimation ${\hat{h}}^{\left(p\right)}$ using Equation (38);(b)Increase h_count; Increase *p*;(c)Check the value of the loop variable *p*: if p<Max_count, continue; otherwise, go to Step4 (3).The inner loop counter variable of object estimation: o_count=0;The iteration process of object estimation, *q*=0, 1, *…*, Max_count(a)The conjugate gradient method was used to optimize Equation (35) and to obtain object image estimation ${\hat{o}}^{\left(q\right)}$;(b)Increase o_count; Increase *q*;(c)Check loop variable *q*: if *q* < Max_count, continue; otherwise, go to Step4 (5);Check whether the outer loop is finished: if *j* > MaxIteration, then go to **Step 5**;Increase *j*, return to Step4 (1).**Step 5**: If j>MaxIteration, then output object estimate image $\hat{o}$ and PSF estimation $\hat{h}$, and end the algorithm; otherwise go to **Step 4**.

## 4. Experimental Results

In this section, we use simulated images and a set of real AO images to verify our proposed restoration algorithm. We present experimental results on AO images taken by a 1.2 m AO telescope (Yunnan Observatory, Yunnan, China) which uses the Hartmann–Shack wavefront sensor from the Chinese Academy of Sciences at Yunnan Observatory on 3 December 2006. The second set of AO images are from the observations of celestial bodies on 13 January 2007. The values of the main parameters for the AO imaging system are shown in Table 1.

In order to verify the restoration effect and the reliability of our algorithm, we implement it using MATLAB 6.5 (The MathWorks, Natick, MA, USA) and test it on a 2.5 GHz Intel i5-2525M CPU (Intel Corporation, Santa Clara, CA, USA) with 4.0 GB of RAM running on a 32 bit Windows 7 operating system (Microsoft Corporation, Redmond, WA, USA).

In order to evaluate the image restoration result, we adopt the objective evaluation criteria Normalized Mean Square Error ($E_{\mathrm{NMSE}}$) [28] and Full Width at Half Maximum ($E_{\mathrm{FWHM}}$) [29] in this paper. The $E_{\mathrm{NMSE}}$ is defined as

(39) $$E_{\mathrm{NMSE}}=\frac{\sum_{x=1}^{N_{1}}\sum_{y=1}^{N_{2}}{\left(\hat{o}\left(x,y\right)-i\left(x,y\right)\right)}^{2}}{\sum_{x=1}^{N_{1}}\sum_{y=1}^{N_{2}}{\left(i\left(x,y\right)\right)}^{2}}.$$

$E_{\mathrm{NMSE}}$ is an estimator of the overall deviations between the original image and the object image. A lower $E_{\mathrm{NMSE}}$ value indicates that the deviation between the original image and the object image is small and a better restored image quality is obtained.

$E_{\mathrm{FWHM}}$ is a parameter commonly used to describe the width of a “bump” on a curve or function. It is given by the distance between points on the curve at which the function reaches half its maximum value. For a point-like object in an astronomical image, $E_{\mathrm{FWHM}}$ is two times that of the pixel distance between the image peak value and half its peak value [29]. It is used to measure for the quality of an image in astronomical observations. The $E_{\mathrm{FWHM}}$ components along the *x*- and *y*-directions of an image are used for evaluation in astronomical observations with the following formula:

(40) $$E_{\mathrm{FWHM}}=\sqrt{E_{{\mathrm{FWHM}}_{x}}^{2}+E_{{\mathrm{FWHM}}_{y}}^{2}},$$

where $E_{{\mathrm{FWHM}}_{x}}$ and $E_{{\mathrm{FWHM}}_{y}}$ represent the peak pixels for the $E_{\mathrm{FWHM}}$ on the *x*- and *y*-directions, respectively. The closer the value of $E_{\mathrm{FWHM}}$ is to the optical diffraction limit for the AO imaging system, the better it is for the image restoration quality.

### 4.1. The Restoration Experiment on Simulated Images

In the simulation experiments in this paper, the original images (“House” with 256 × 256 pixels, “Chemical Plant” with 256 × 256 pixels, and “The Little Girl” with 256 × 256 pixels) are selected from [30]. Ten frames of simulation degraded images are generated by image degradation software from the Key Optical Laboratory of the Chinese Academy of Sciences [25], and then Gaussian white noise is added and the signal-to-noise ratio (SNR) of the images is set to be 20 dB, 25 dB, and 30 dB with real AO imaging conditions including atmospheric turbulence. The equivalent parameters for the four layers turbulence model are the same as those in Reference [22], and we set the parameters to be the same as the 1.2 m AO telescope at the observatory in Yunnan, China. The main parameters of the telescope imaging system are the atmospheric coherence as shown in Table 1. The simulated degraded images are assumed to be at certain distances so that one arcsecond imaging angle corresponds to degradation of a certain linear scale. Experiments on the ML-EM algorithm [10], the CPF-adaptive algorithm [11], the RT-IEM algorithm [12], the VBBD-TV algorithm [9], and our algorithm are compared.

**Figure 1:**
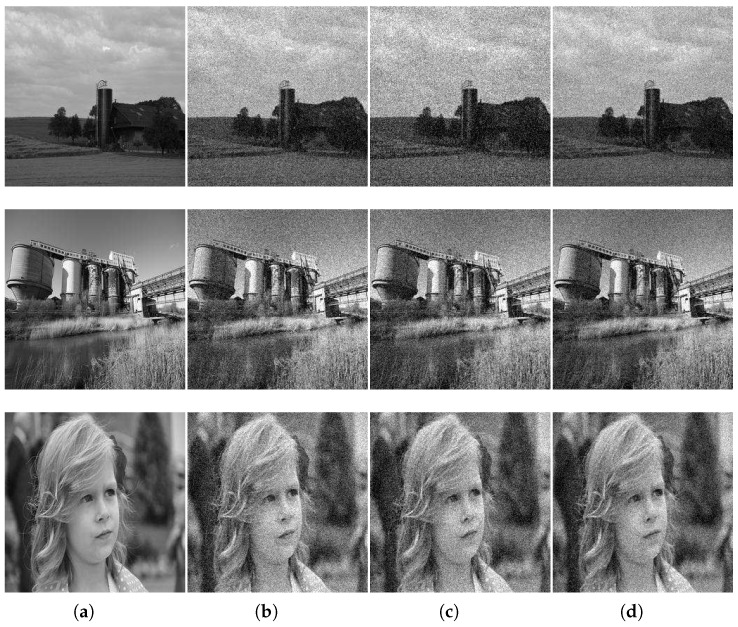
The original images and the simulated multi-frame degradation images. (**a**) input images of the datasets; (**b**) images degraded by the 5 × 5 PSF at SNR of 20 dB under ideal conditions; (**c**) images degraded by the 5 × 5 PSF at SNR of 25 dB under ideal conditions; (**d**) images degraded by the 5 × 5 PSF at SNR of 30 dB under ideal conditions.

**Figure 2:**
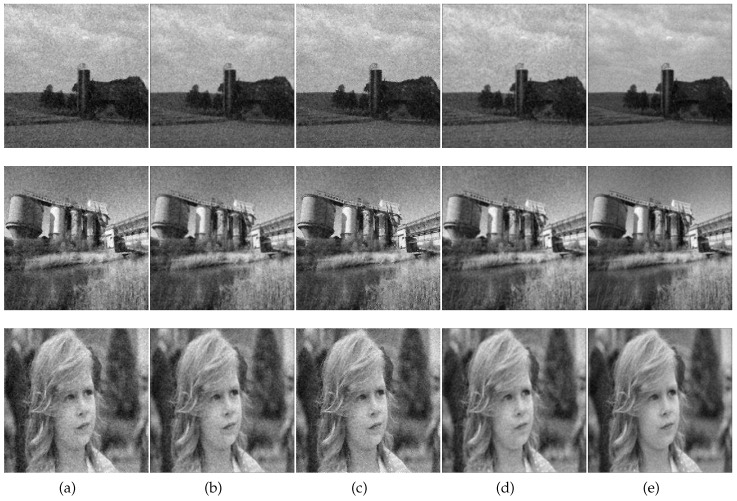
The comparison results of the restored images based on four algorithms (with 300 iterations). (**a**) by the ML-EM algorithm; (**b**) by the CPF-adaptive algorithm; (**c**) by the RT-IEM algorithm; (**d**) by the VBBD-TV algorithm; (**e**) by our algorithm with β=1.31 and ξ=1.35.

Figure 1 is the original image and simulated multi-frame degradation images. Figure 1a is the original images, and example frames with turbulence and noise are shown in Figure 1b–d (to save space, only three frames are shown). The comparison results based on the five algorithms are shown in Figure 2. In our algorithm, the parameters β=1.31 and ξ=1.35 were selected experimentally for visually acceptable results. Table 2 gives the results of our algorithm and those of the ML-EM [10], CPF-adaptive [11], RT-IEM [12], and VBBD-TV [9] methods, and the number of iterations for the four algorithms is 300. Our algorithm ranks on the top of the list, and the results demonstrate the superiority of our method.

### 4.2. Restoration Experiments on Binary-Star AO Images

For adaptive optics compensated images, the degradation is dominated by the residual fine-scale wavefront error, which requires several parameters to characterize and varies from frame to frame. Now, we show the restoration results of our proposed algorithm on binary-star AO images that were taken by a 1.2 m AO imaging system on 3 December 2006. The adaptive optics bench is a system with 88 actuators, with a Shack–Hartman wave-front sensor (64 sub-apertures). The values of the main parameters for the AO imaging system are shown in Table 1. The estimated PSF model for an AO image is under the following conditions: the full field-of-view for the system is $20^{\prime \prime }$; the Zernike model for the fully corrected turbulence effect is with the first 35 orders; the field-of-view is $10^{\prime \prime }$; the size of the space-variant PSF is 5×5 pixels; and the isoplanatic angle θ is $2^{\prime \prime }$. The AO image restoration experiments based on the ML-EM algorithm [10], the CPF-adaptive algorithm [11], the RT-IEM algorithm [12], the VBBD-TV algorithm [9], and our algorithm are compared.

**Figure 3:**
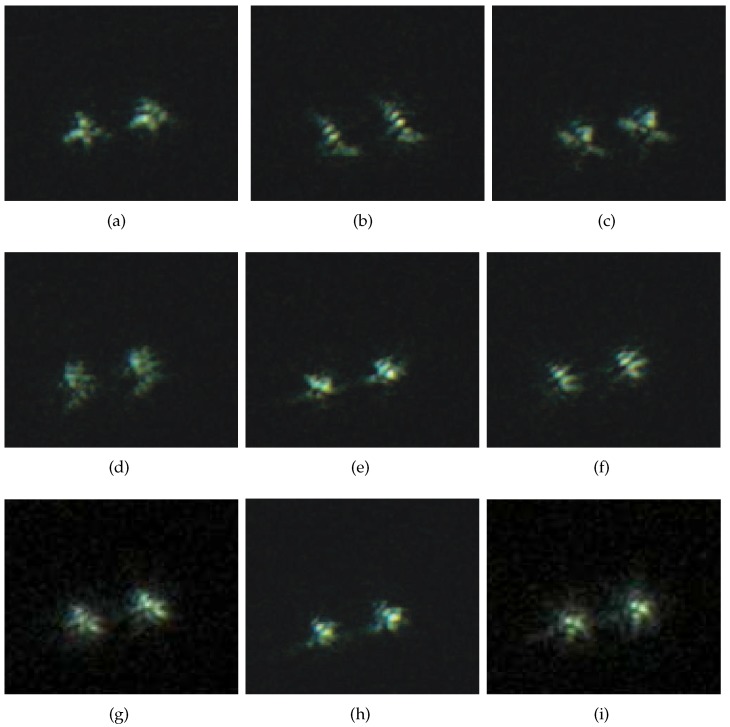
Nine frames of binary-star images by an AO system and their variances. (**a**) Frame 1 with variance $S_{1}^{2}=536.34$; (**b**) Frame 2 with variance $S_{2}^{2}=518.06$; (**c**) Frame 3 with variance $S_{3}^{2}=566.23$; (**d**) Frame 4 with variance $S_{4}^{2}=495.07$; (**e**) Frame 5 with variance $S_{5}^{2}=552.10$; (**f**) Frame 6 with variance $S_{6}^{2}=545.26$; (**g**) Frame 7 with variance $S_{7}^{2}=336.34$; (**h**) Frame 8 with variance $S_{8}^{2}=522.95$; (**i**) Frame 9 with variance $S_{9}^{2}=530.72$.

We apply our frame select technique, which is introduced in Section 2.2, and select 50 frames from 200 of the observed AO images as the input images for blind convolution. Figure 3 shows the observed multi-frame degraded binary-star AO images (only nine frames are shown). In order to obtain better restoration results, our frame selection technique is used to select a group of high-quality degraded AO images to improve the stability of the algorithm.

In Figure 3, frame selection experiment based on image variance is carried out for binary-star AO images. The variance of each degraded image is calculated using Equation (5). The size of the binary-star AO image is 132×121 pixels.

The variance value for each observed AO image is given in the caption of Figure 3. The variance values show that most of the variance values for the images are close to each other, and their values are between 500 and 570. While affected by Gaussian noise, the variance of the 3rd frame is 566.23, which is higher than the others. The variance of the 7th frame is influenced by the PSF blurring effect and it is 336.34, so its value is much lower than that of the other images. Therefore, the 3rd and 7th frames should be eliminated for estimation purposes.

**Figure 4:**
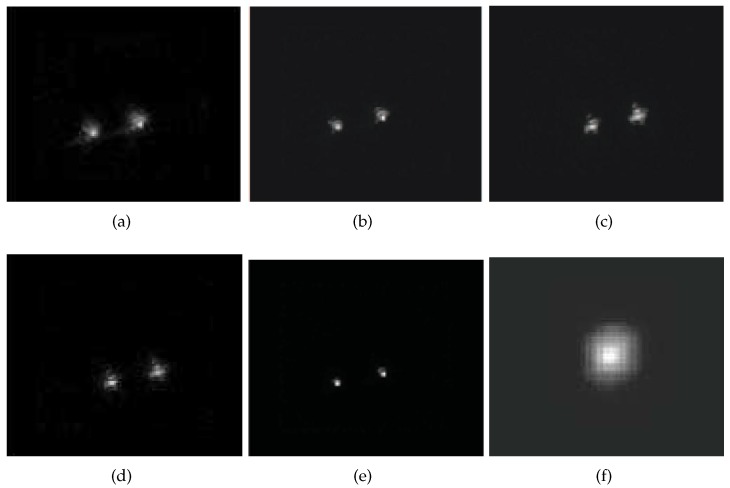
The restoration results comparison on multi-frames binary-star AO images. (**a**) restored image by the ML-EM algorithm; (**b**) restored image by the CPF-adaptive algorithm; (**c**) restored image by the RT-IEM algorithm; (**d**) restored image by the VBBD-TV algorithm; (**e**) restored image by our algorithm; (**f**) estimated PSF by our algorithm.

To test the performance of our proposed algorithm, image restoration experiments were carried out, and the selected AO images for the experiments were shown in Figure 3 (Frames 1, 2, 4, 5, 6, 8, 9 were selected in the experiment). We choose β=1.23, and the parameter for the regularization function is ξ=1.35 with 300 iterations. Figure 4 shows the restoration results on degraded AO images based on five algorithms. Figure 4a is the restored image based on the ML-EM algorithm with $E_{\mathrm{NMSE}}=0.0413$ and $E_{\mathrm{FWHM}}=5.73$; Figure 4b is the restored image based on the CPF-adaptive algorithm with $E_{\mathrm{NMSE}}=0.0386$ and $E_{\mathrm{FWHM}}=5.97$; Figure 4c is the restored image based on the RT-IEM algorithm with $E_{\mathrm{NMSE}}=0.0363$ and $E_{\mathrm{FWHM}}=5.99$; Figure 4d is the restored image based on the VBBD-TV algorithm with $E_{\mathrm{NMSE}}=0.0358$ and $E_{\mathrm{FWHM}}=5.87$; and Figure 4e is the restored image based on our algorithm with $E_{\mathrm{NMSE}}=0.0301$ and $E_{\mathrm{orFWHM}}=6.13$. The total number of iteration is 200, and the restoration results are very close to the diffraction limit of the 1.2 m AO telescope. Figure 4f is the estimation of the PSF based on our algorithm.

In order to verify our algorithm, we compare our algorithm with four other algorithms (the ML-EM, CPF-adaptive, RT-IEM, and VBBD-TV algorithms) for the binary-star images, and the objective evaluation criteria for the experimental results are measured by $E_{\mathrm{NMSE}}$, $E_{\mathrm{FWHM}}$, and the computation time, which are shown in Table 3, and the number of iterations for the five algorithms is 300.

Comparing with the ML-EM, CPF-adaptive, RT-IEM, and VBBD-TV algorithms, we can see that the $E_{\mathrm{NMSE}}$ measures from our algorithm are decreased by 19%, 4.2%, 2.8%, and 7.7%, respectively. It is shown in Table 3 that our algorithm can obtain a $E_{\mathrm{FWHM}}$, which is closer to the diffraction limit of the AO system and can obtain lower $E_{\mathrm{NMSE}}$ measures. Therefore, our proposed method can restore binary-star degraded images effectively where the degradation was due to the effect of atmospheric turbulence acting on the optical telescope. The computation load of our method is slightly higher than the other four restoration algorithms, and we plan to further improve the performance of our algorithm in the future.

**Figure 5:**
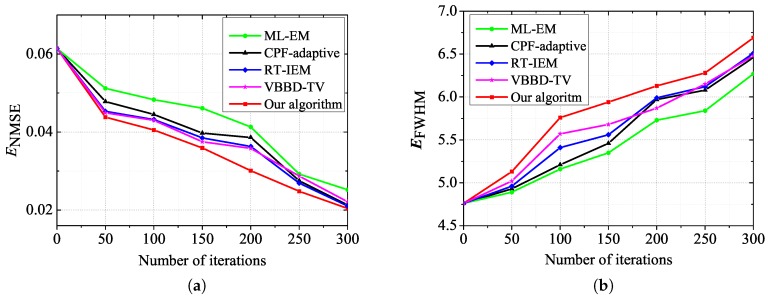
The restoration comparisons on $E_{\mathrm{NMSE}}$ and $E_{\mathrm{FWHM}}$ measures versus the iteration number for the five methods. (**a**) $E_{\mathrm{NMSE}}$ versus the iteration number of the five algorithms; (**b**) $E_{\mathrm{FWHM}}$ versus the iteration number of the five algorithms.

Moreover, the $E_{\mathrm{NMSE}}$ and $E_{\mathrm{FWHM}}$ measures of the best restored images by the five algorithms are compared in Figure 5. The $E_{\mathrm{NMSE}}$ measure versus the iteration number for the five algorithms on the binary-star AO images are plotted in Figure 5a. Figure 5b is the $E_{\mathrm{FWHM}}$ results for the five restoration algorithms. It can be seen from Figure 5b that our algorithm has $E_{\mathrm{FWHM}}$ values that are close to the diffraction limit of the AO system when applied to binary-star images. This shows that our restoration algorithm can effectively restore the degraded AO images.

### 4.3. Sensitivity Analysis

**Figure 6:**
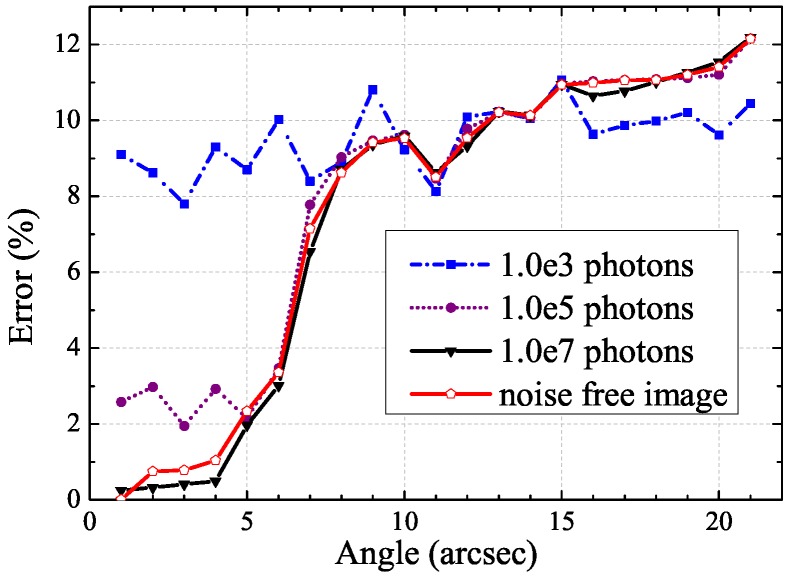
Error on the estimation of the PSF model in the case of a noise free image, and in the cases of photon noises with $10^{3}$, $10^{5}$, and $10^{7}$ photons in the whole image, respectively.

Sensitivity analysis is the study of how the uncertainty in the output of a model can be portioned to different sources of variation in the model inputs [31]. In order to evaluate the benefits of using the proposed PSF initial estimation, the computation of the sensitivity indices of a PSF model was performed. In an AO image restoration process, we initialize the restoration algorithm with a perfect PSF. In this work, extensive simulations are performed to test the behaviour of the proposed PSF estimation. In a realistic case, the degraded AO image is affected by the turbulence effect, the photon, and detector noise. We assume that the dominant noise is the photon noise. The simulation conditions are the followings: the simulation is performed on three different photon noise levels varying in: $10^{3}$ photons, $10^{5}$ photons, and $10^{7}$ photons; and the angle θ varies from 1 arcsec for the minimum value to 21 arcsec for the maximum value. Figure 6 shows the error on the estimation of the PSF model in the case of a noise free image, and in the cases of photon noises with $10^{3}$, $10^{5}$, and $10^{7}$ photons in the whole image, respectively. We plot in Figure 6 the errors which are given in percentages on the magnitude estimation of star images using an isoplanatic deconvolution process. In the case of an isoplanatic deconvolution, the PSF is constant in the whole field of view [21]. For a given noise level, a limit angle θ can be defined below when the error is lower than the noise error. The lower the noise level, the greater the limit angle (see Figure 6). For instance, the error becomes greater than the noise error in the case of $10^{3}$ photons for angle θ≥7. This limit angle can be used to define an isoplanatic angle for our AO image restoration method.

Furthermore, we perform a sensitivity analysis on the PSF starting values for our proposed AO image restoration algorithm. To validate the sensitivity behaviour of the PSF model, the experiment is performed and the conditions are:

- Noise root mean square (RMS) changes from one percent for the minimum value of the image to 20 percent for the maximum value for the image;
- Fifty noise realizations are calculated for each RMS noise value;
- The simulation is performed on three different sub-images varying in size: a 64×64 pixels central region of the image, a 128×128 pixels central region of the image, and the whole 256×256 pixels image.

**Figure 7:**
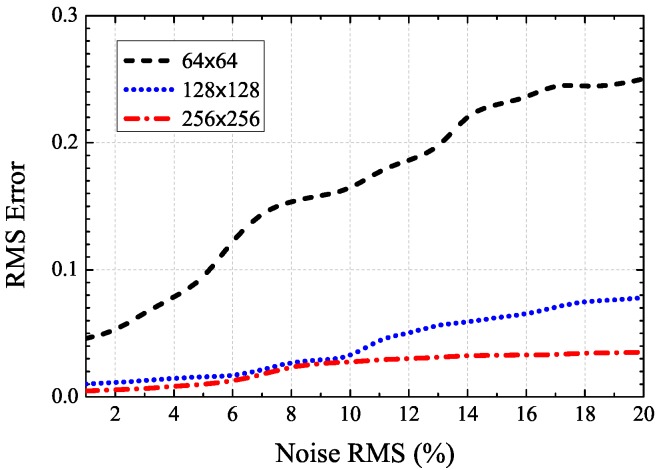
RMS error on estimation of the PSF initial value for different noise levels (in percentage).

Figure 7 shows the RMS error on the estimation of the initial PSF values for different levels of noise and varying data size. It indicates that the initial value of our PSF estimation is useful to provide less sensitive final PSF estimation in our proposed restoration algorithm. This simulation shows that the PSF estimator exhibits a lower sensitivity to noises, which opens the way to its use for real AO images.

## 5. Conclusions

This paper presents a novel adaptive optics images restoration algorithm based on our frame selection method and the PSF model for the turbulence effect. There are two main contributions in this paper. The proposed frame selection method provides a reasonable criterion to evaluate the quality of a degraded AO image based on the variance of the image. We also propose a PSF reconstruction method for AO images, which can accurately estimate the PSF model in a blind deconvolution process using the initial estimate value. A theoretical expression of the PSF is derived. According to the Poisson distribution model for establishing a joint log-likelihood function for the multi-frame AO images, the proposed algorithm for the maximum likelihood estimator is used for the object image and the PSF estimation for each frame. A series of experiments were carried out to verify the effectiveness of the proposed algorithm and show its superiority over the current state-of-the-art blind deconvolution methods. The comparisons with the ML-EM, CPF-adaptive, RT-IEM, and VBBD-TV methods also indicate that our proposed algorithm is competitive on AO image restoration. Future works should focus on reducing the computation time by employing parallel computing. Our proposed algorithm can be used for restoring observed AO images, and it has the potential for real applications.

## Figures and Tables

**Table 1:** The values of the main parameters for an AO imaging system.

Parameter Name	Parameter Value	Remarks
$r_{0}$	13 cm	Atmospheric coherence length
λ	0.72 μm	Central wavelength
*f*	20 m	Imaging focal length
*D*	1.03 m	Telescope aperture
The size for imaging CCD	320 × 240 pixel	
Size of pixel in CCD	6.7 μm	
Imaging observation range	0.7−0.9 μm	
Field of view for imaging system	$24^{\prime \prime }\times 18^{\prime \prime }$	

**Table 2:** Comparison results on $E_{\mathrm{NMSE}}$ and running time of different restoration algorithms.

		ML-EM			CPF-Adaptive			RT-IEM			VBBD-TV			Our Algorithm	
Image Names		$E_{\mathrm{NMSE}}$	Running Time (s)		$E_{\mathrm{NMSE}}$	Running Time (s)		$E_{\mathrm{NMSE}}$	Running Time (s)		$E_{\mathrm{NMSE}}$	Running Time (s)		$E_{\mathrm{NMSE}}$	Running Time (s)
House		0.0034	12.18		0.0025	13.27		0.0030	13.96		0.0023	13.87		0.0015	13.90
Chemical Plant		0.0069	11.97		0.0072	12.89		0.0054	13.04		0.0051	13.12		0.0045	13.21
The Little Girl		0.0046	9.24		0.0039	10.54		0.0028	10.91		0.0021	10.87		0.0017	11.08

**Table 3:** The $E_{\mathrm{NMSE}}$, $E_{\mathrm{FWHM}}$, and the computation time comparison for the five algorithms for binary-star images restoration (iteration number is 300).

Algorithms	$E_{\mathrm{NMSE}}$	$E_{\mathrm{FWHM}}$ (pixel)	Computation Time (s)
ML-EM	0.0252	6.27	9.872
CPF-adaptive	0.0213	6.46	12.196
RT-IEM	0.0210	6.51	10.983
VBBD-TV	0.0221	6.48	8.624
Our algorithm	0.0204	6.69	12.257
